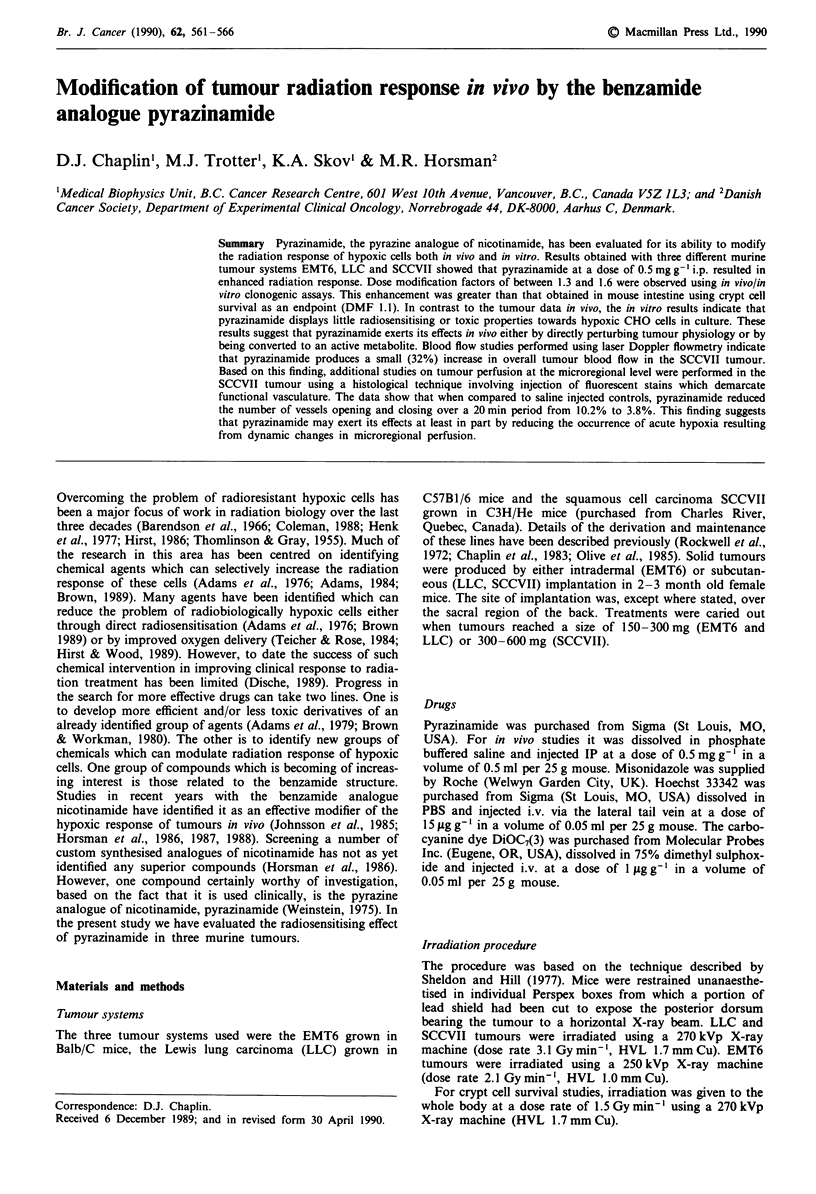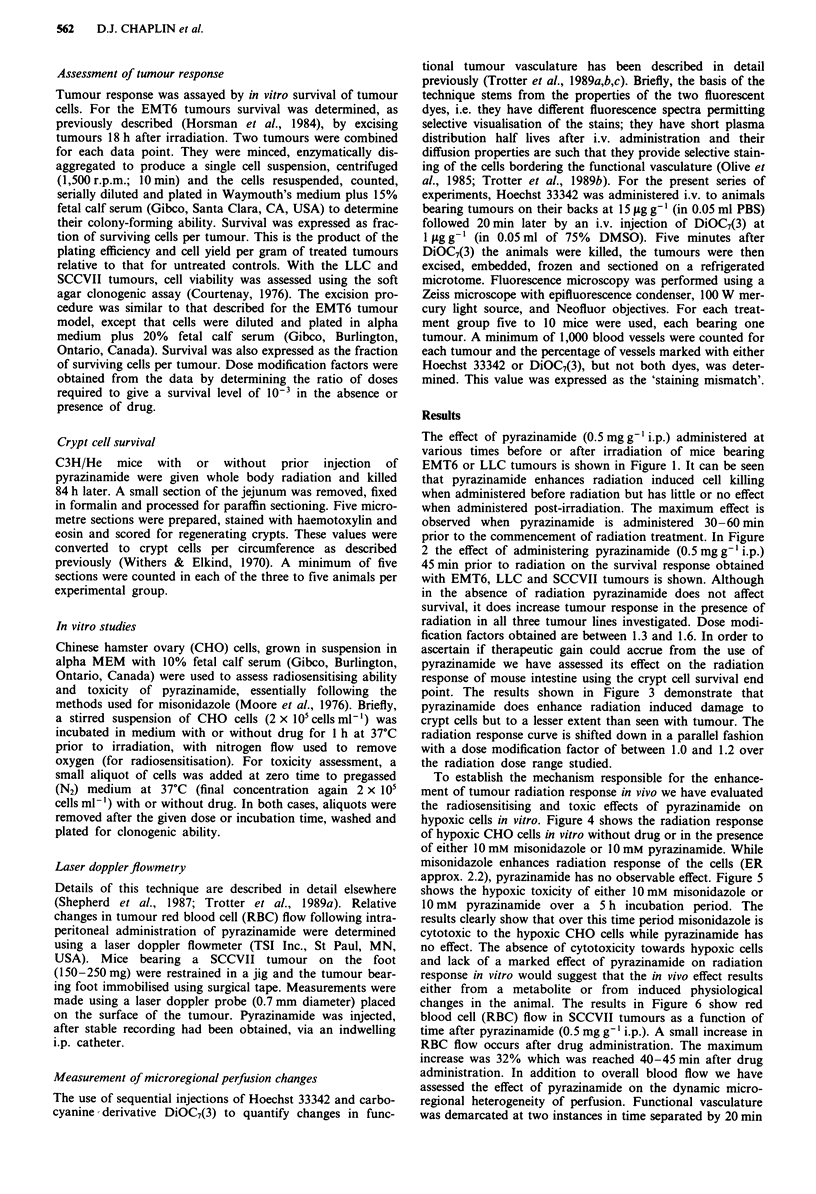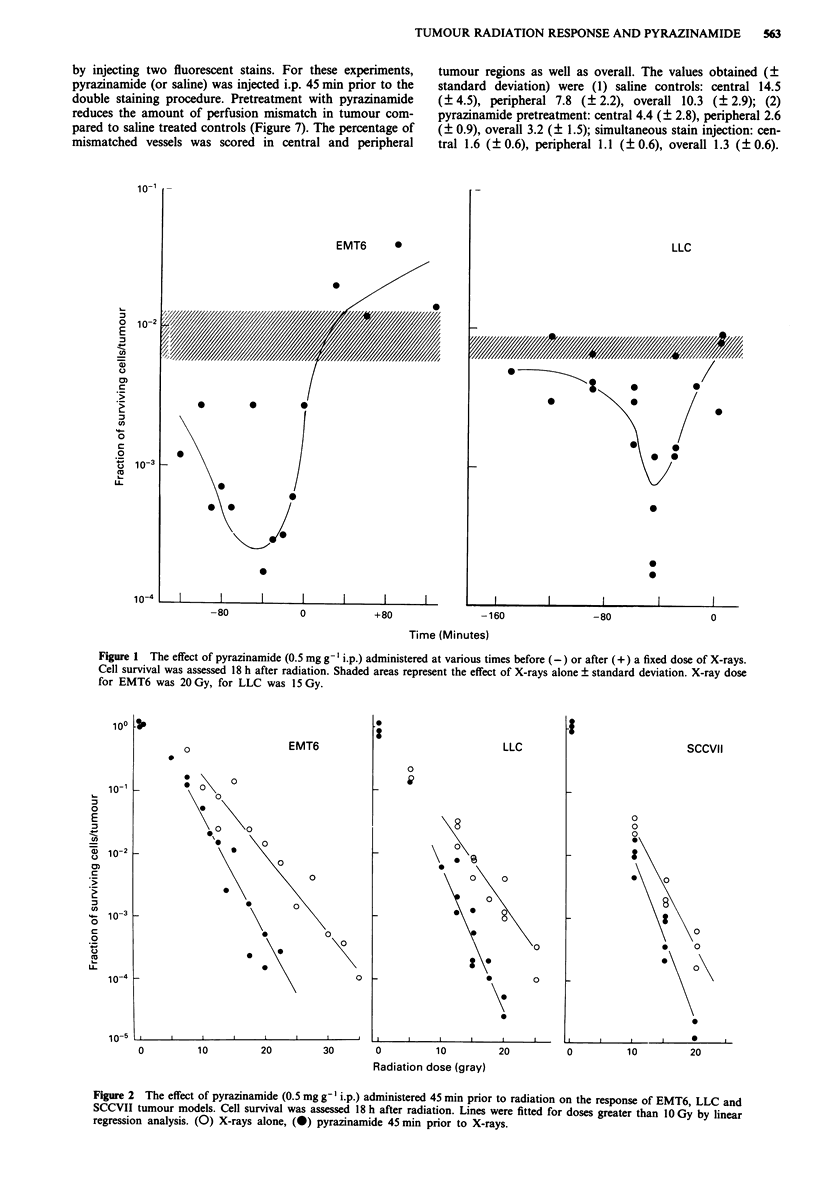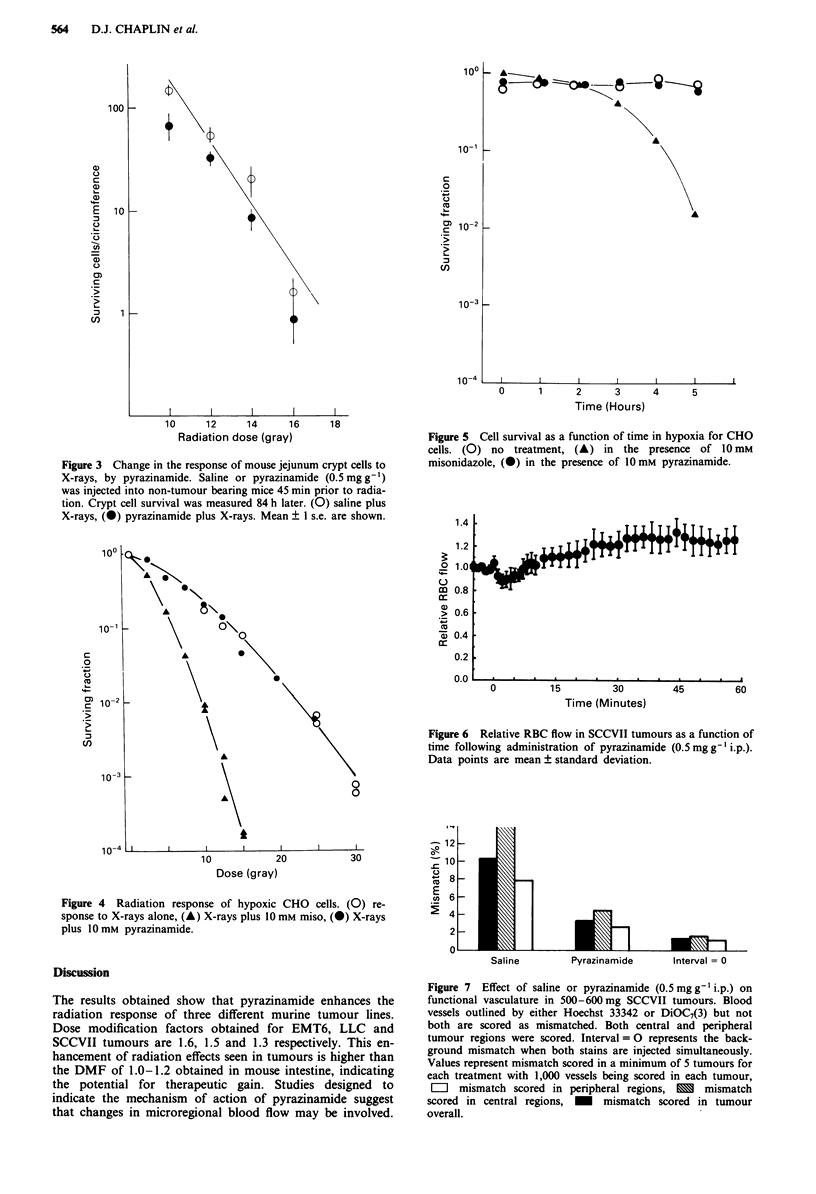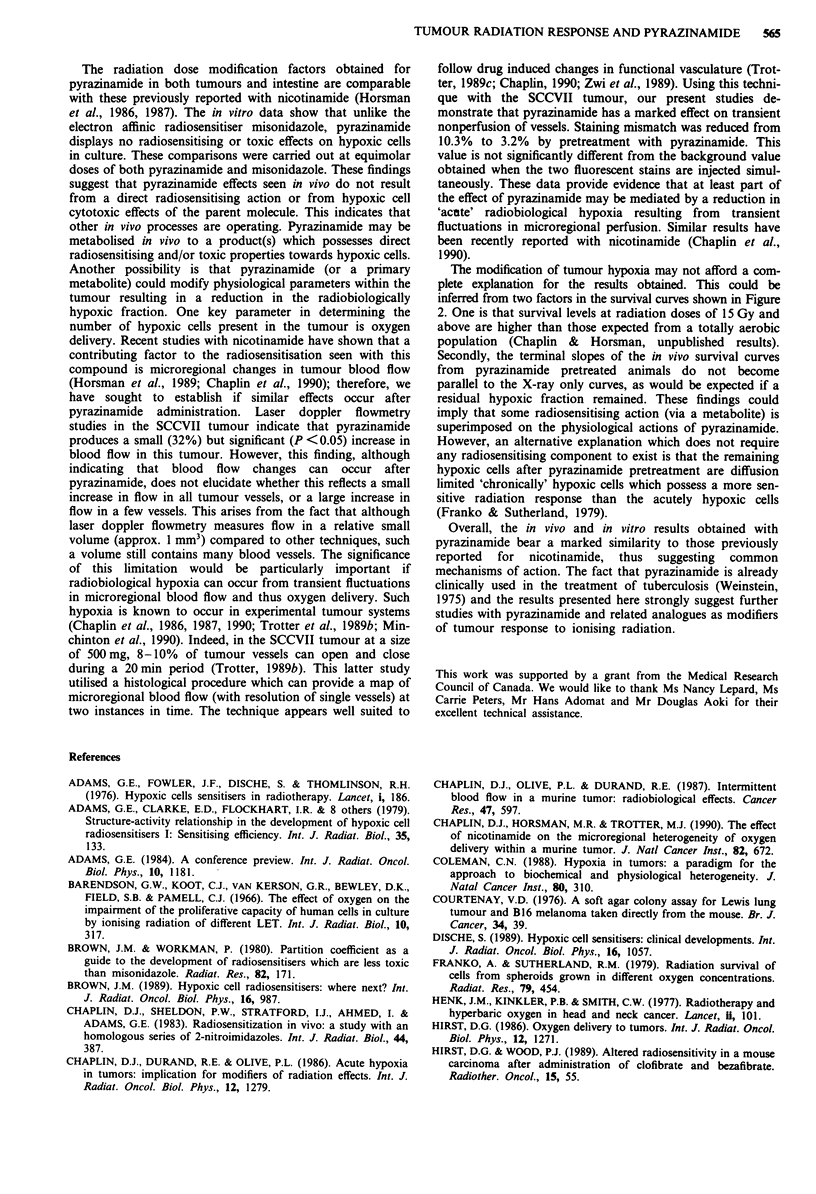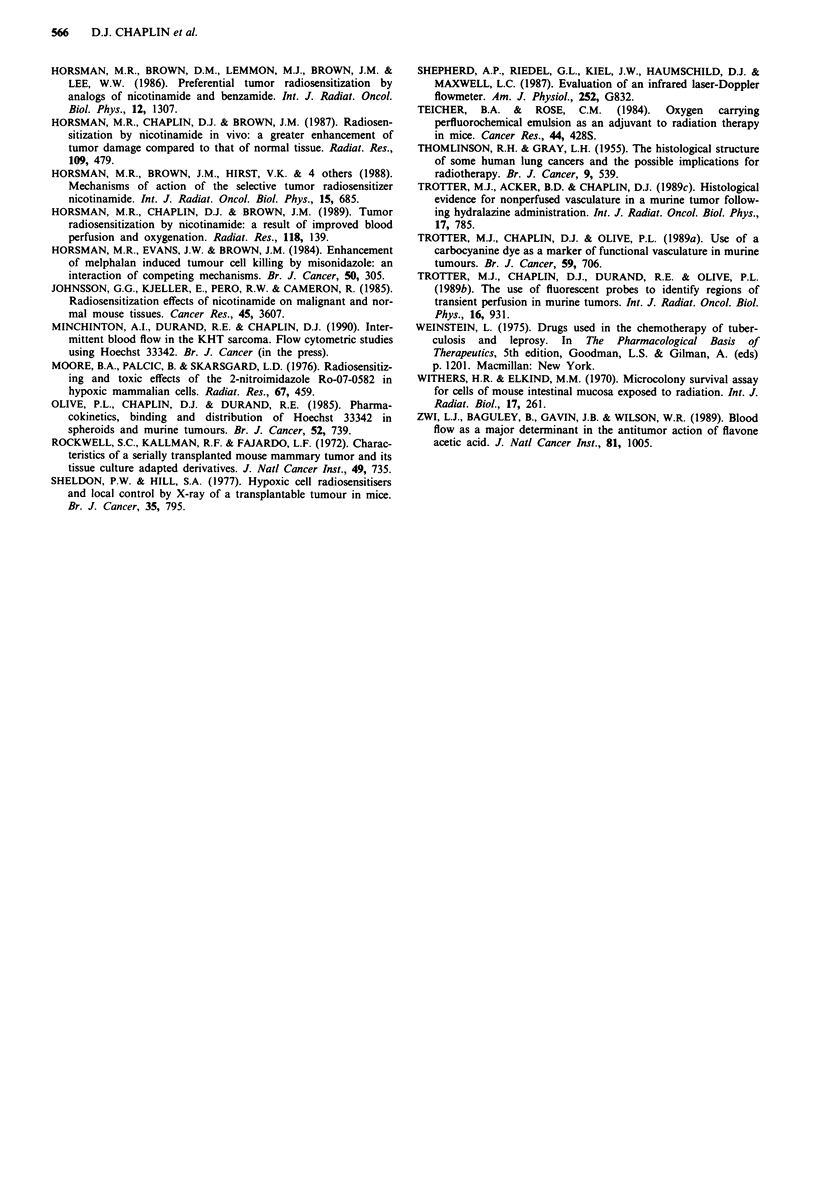# Modification of tumour radiation response in vivo by the benzamide analogue pyrazinamide.

**DOI:** 10.1038/bjc.1990.330

**Published:** 1990-10

**Authors:** D. J. Chaplin, M. J. Trotter, K. A. Skov, M. R. Horsman

**Affiliations:** Medical Biophysics Unit, B.C. Cancer Research Centre, Vancouver, Canada.

## Abstract

Pyrazinamide, the pyrazine analogue of nicotinamide, has been evaluated for its ability to modify the radiation response of hypoxic cells both in vivo and in vitro. Results obtained with three different murine tumour systems EMT6, LLC and SCCVII showed that pyrazinamide at a dose of 0.5 mg g-1 i.p. resulted in enhanced radiation response. Dose modification factors of between 1.3 and 1.6 were observed using in vivo/in vitro clonogenic assays. This enhancement was greater than that obtained in mouse intestine using crypt cell survival as an endpoint (DMF 1.1). In contrast to the tumour data in vivo, the in vitro results indicate that pyrazinamide displays little radiosensitising or toxic properties towards hypoxic CHO cells in culture. These results suggest that pyrazinamide exerts its effects in vivo either by directly perturbing tumour physiology or by being converted to an active metabolite. Blood flow studies performed using laser Doppler flowmetry indicate that pyrazinamide produces a small (32%) increase in overall tumour blood flow in the SCCVII tumour. Based on this finding, additional studies on tumour perfusion at the microregional level were performed in the SCCVII tumour using a histological technique involving injection of fluorescent stains which demarcate functional vasculature. The data show that when compared to saline injected controls, pyrazinamide reduced the number of vessels opening and closing over a 20 min period from 10.2% to 3.8%. This finding suggests that pyrazinamide may exert its effects at least in part by reducing the occurrence of acute hypoxia resulting from dynamic changes in microregional perfusion.


*Br. J. Cancer* (1990), **62**, 561–566 © Macmillan Press Ltd., 1990

### Modification of tumour radiation response *in vivo* by the benzamide analogue pyrazinamide

D.J. Chaplin[1], M.J. Trotter[1], K.A. Skov[1] & M.R. Horsman[2]

[1]*Medical Biophysics Unit, B.C. Cancer Research Centre, 601 West 10th Avenue, Vancouver, B.C., Canada V5Z 1L3; and* [2]*Danish Cancer Society, Department of Experimental Clinical Oncology, Norrebrogade 44, DK-8000, Aarhus C, Denmark.*

**Summary** Pyrazinamide, the pyrazine analogue of nicotinamide, has been evaluated for its ability to modify the radiation response of hypoxic cells both *in vivo* and *in vitro*. Results obtained with three different murine tumour systems EMT6, LLC and SCCVII showed that pyrazinamide at a dose of 0.5 mg g$^{-1}$ i.p. resulted in enhanced radiation response. Dose modification factors of between 1.3 and 1.6 were observed using *in vivo/in vitro* clonogenic assays. This enhancement was greater than that obtained in mouse intestine using crypt cell survival as an endpoint (DMF 1.1). In contrast to the tumour data *in vivo*, the *in vitro* results indicate that pyrazinamide displays little radiosensitising or toxic properties towards hypoxic CHO cells in culture. These results suggest that pyrazinamide exerts its effects *in vivo* either by directly perturbing tumour physiology or by being converted to an active metabolite. Blood flow studies performed using laser Doppler flowmetry indicate that pyrazinamide produces a small (32%) increase in overall tumour blood flow in the SCCVII tumour. Based on this finding, additional studies on tumour perfusion at the microregional level were performed in the SCCVII tumour using a histological technique involving injection of fluorescent stains which demarcate functional vasculature. The data show that when compared to saline injected controls, pyrazinamide reduced the number of vessels opening and closing over a 20 min period from 10.2% to 3.8%. This finding suggests that pyrazinamide may exert its effects at least in part by reducing the occurrence of acute hypoxia resulting from dynamic changes in microregional perfusion.

Overcoming the problem of radioresistant hypoxic cells has been a major focus of work in radiation biology over the last three decades (Barendson *et al.*, 1966; Coleman, 1988; Henk *et al.*, 1977; Hirst, 1986; Thomlinson & Gray, 1955). Much of the research in this area has been centred on identifying chemical agents which can selectively increase the radiation response of these cells (Adams *et al.*, 1976; Adams, 1984; Brown, 1989). Many agents have been identified which can reduce the problem of radiobiologically hypoxic cells either through direct radiosensitisation (Adams *et al.*, 1976; Brown 1989) or by improved oxygen delivery (Teicher & Rose, 1984; Hirst & Wood, 1989). However, to date the success of such chemical intervention in improving clinical response to radiation treatment has been limited (Dische, 1989). Progress in the search for more effective drugs can take two lines. One is to develop more efficient and/or less toxic derivatives of an already identified group of agents (Adams *et al.*, 1979; Brown & Workman, 1980). The other is to identify new groups of chemicals which can modulate radiation response of hypoxic cells. One group of compounds which is becoming of increasing interest is those related to the benzamide structure. Studies in recent years with the benzamide analogue nicotinamide have identified it as an effective modifier of the hypoxic response of tumours *in vivo* (Johnsson *et al.*, 1985; Horsman *et al.*, 1986, 1987, 1988). Screening a number of custom synthesised analogues of nicotinamide has not as yet identified any superior compounds (Horsman *et al.*, 1986). However, one compound certainly worthy of investigation, based on the fact that it is used clinically, is the pyrazine analogue of nicotinamide, pyrazinamide (Weinstein, 1975). In the present study we have evaluated the radiosensitising effect of pyrazinamide in three murine tumours.

### Materials and methods

### *Tumour systems*

The three tumour systems used were the EMT6 grown in Balb/C mice, the Lewis lung carcinoma (LLC) grown in C57Bl/6 mice and the squamous cell carcinoma SCCVII grown in C3H/He mice (purchased from Charles River, Quebec, Canada). Details of the derivation and maintenance of these lines have been described previously (Rockwell *et al.*, 1972; Chaplin *et al.*, 1983; Olive *et al.*, 1985). Solid tumours were produced by either intradermal (EMT6) or subcutaneous (LLC, SCCVII) implantation in 2–3 month old female mice. The site of implantation was, except where stated, over the sacral region of the back. Treatments were caried out when tumours reached a size of 150–300 mg (EMT6 and LLC) or 300–600 mg (SCCVII).

### *Drugs*

Pyrazinamide was purchased from Sigma (St Louis, MO, USA). For *in vivo* studies it was dissolved in phosphate buffered saline and injected IP at a dose of 0.5 mg g$^{-1}$ in a volume of 0.5 ml per 25 g mouse. Misonidazole was supplied by Roche (Welwyn Garden City, UK). Hoechst 33342 was purchased from Sigma (St Louis, MO, USA) dissolved in PBS and injected i.v. via the lateral tail vein at a dose of 15 $\mu$g g$^{-1}$ in a volume of 0.05 ml per 25 g mouse. The carbocyanine dye DiOC$_7$(3) was purchased from Molecular Probes Inc. (Eugene, OR, USA), dissolved in 75% dimethyl sulphoxide and injected i.v. at a dose of 1 $\mu$g g$^{-1}$ in a volume of 0.05 ml per 25 g mouse.

### *Irradiation procedure*

The procedure was based on the technique described by Sheldon and Hill (1977). Mice were restrained unanaesthetised in individual Perspex boxes from which a portion of lead shield had been cut to expose the posterior dorsum bearing the tumour to a horizontal X-ray beam. LLC and SCCVII tumours were irradiated using a 270 kVp X-ray machine (dose rate 3.1 Gy min$^{-1}$, HVL 1.7 mm Cu). EMT6 tumours were irradiated using a 250 kVp X-ray machine (dose rate 2.1 Gy min$^{-1}$, HVL 1.0 mm Cu).

For crypt cell survival studies, irradiation was given to the whole body at a dose rate of 1.5 Gy min$^{-1}$ using a 270 kVp X-ray machine (HVL 1.7 mm Cu).

Correspondence: D.J. Chaplin.
Received 6 December 1989; and in revised form 30 April 1990.

*Assessment of tumour response*

Tumour response was assayed by *in vitro* survival of tumour cells. For the EMT6 tumours survival was determined, as previously described (Horsman *et al.*, 1984), by excising tumours 18 h after irradiation. Two tumours were combined for each data point. They were minced, enzymatically disaggregated to produce a single cell suspension, centrifuged (1,500 r.p.m.; 10 min) and the cells resuspended, counted, serially diluted and plated in Waymouth's medium plus 15% fetal calf serum (Gibco, Santa Clara, CA, USA) to determine their colony-forming ability. Survival was expressed as fraction of surviving cells per tumour. This is the product of the plating efficiency and cell yield per gram of treated tumours relative to that for untreated controls. With the LLC and SCCVII tumours, cell viability was assessed using the soft agar clonogenic assay (Courtenay, 1976). The excision procedure was similar to that described for the EMT6 tumour model, except that cells were diluted and plated in alpha medium plus 20% fetal calf serum (Gibco, Burlington, Ontario, Canada). Survival was also expressed as the fraction of surviving cells per tumour. Dose modification factors were obtained from the data by determining the ratio of doses required to give a survival level of $10^{-3}$ in the absence or presence of drug.

*Crypt cell survival*

C3H/He mice with or without prior injection of pyrazinamide were given whole body radiation and killed 84 h later. A small section of the jejunum was removed, fixed in formalin and processed for paraffin sectioning. Five micrometre sections were prepared, stained with haemotoxylin and eosin and scored for regenerating crypts. These values were converted to crypt cells per circumference as described previously (Withers & Elkind, 1970). A minimum of five sections were counted in each of the three to five animals per experimental group.

*In vitro studies*

Chinese hamster ovary (CHO) cells, grown in suspension in alpha MEM with 10% fetal calf serum (Gibco, Burlington, Ontario, Canada) were used to assess radiosensitising ability and toxicity of pyrazinamide, essentially following the methods used for misonidazole (Moore *et al.*, 1976). Briefly, a stirred suspension of CHO cells ($2 \times 10^5$ cells $ml^{-1}$) was incubated in medium with or without drug for 1 h at 37°C prior to irradiation, with nitrogen flow used to remove oxygen (for radiosensitisation). For toxicity assessment, a small aliquot of cells was added at zero time to pregassed ($N_2$) medium at 37°C (final concentration again $2 \times 10^5$ cells $ml^{-1}$) with or without drug. In both cases, aliquots were removed after the given dose or incubation time, washed and plated for clonogenic ability.

*Laser doppler flowmetry*

Details of this technique are described in detail elsewhere (Shepherd *et al.*, 1987; Trotter *et al.*, 1989*a*). Relative changes in tumour red blood cell (RBC) flow following intraperitoneal administration of pyrazinamide were determined using a laser doppler flowmeter (TSI Inc., St Paul, MN, USA). Mice bearing a SCCVII tumour on the foot (150–250 mg) were restrained in a jig and the tumour bearing foot immobilised using surgical tape. Measurements were made using a laser doppler probe (0.7 mm diameter) placed on the surface of the tumour. Pyrazinamide was injected, after stable recording had been obtained, via an indwelling i.p. catheter.

*Measurement of microregional perfusion changes*

The use of sequential injections of Hoechst 33342 and carbocyanine derivative $DiOC_7(3)$ to quantify changes in functional tumour vasculature has been described in detail previously (Trotter *et al.*, 1989*a,b,c*). Briefly, the basis of the technique stems from the properties of the two fluorescent dyes, i.e. they have different fluorescence spectra permitting selective visualisation of the stains; they have short plasma distribution half lives after i.v. administration and their diffusion properties are such that they provide selective staining of the cells bordering the functional vasculature (Olive *et al.*, 1985; Trotter *et al.*, 1989*b*). For the present series of experiments, Hoechst 33342 was administered i.v. to animals bearing tumours on their backs at 15 $\mu g\ g^{-1}$ (in 0.05 ml PBS) followed 20 min later by an i.v. injection of $DiOC_7(3)$ at 1 $\mu g\ g^{-1}$ (in 0.05 ml of 75% DMSO). Five minutes after $DiOC_7(3)$ the animals were killed, the tumours were then excised, embedded, frozen and sectioned on a refrigerated microtome. Fluorescence microscopy was performed using a Zeiss microscope with epifluorescence condenser, 100 W mercury light source, and Neofluor objectives. For each treatment group five to 10 mice were used, each bearing one tumour. A minimum of 1,000 blood vessels were counted for each tumour and the percentage of vessels marked with either Hoechst 33342 or $DiOC_7(3)$, but not both dyes, was determined. This value was expressed as the 'staining mismatch'.

### Results

The effect of pyrazinamide (0.5 mg $g^{-1}$ i.p.) administered at various times before or after irradiation of mice bearing EMT6 or LLC tumours is shown in Figure 1. It can be seen that pyrazinamide enhances radiation induced cell killing when administered before radiation but has little or no effect when administered post-irradiation. The maximum effect is observed when pyrazinamide is administered 30–60 min prior to the commencement of radiation treatment. In Figure 2 the effect of administering pyrazinamide (0.5 mg $g^{-1}$ i.p.) 45 min prior to radiation on the survival response obtained with EMT6, LLC and SCCVII tumours is shown. Although in the absence of radiation pyrazinamide does not affect survival, it does increase tumour response in the presence of radiation in all three tumour lines investigated. Dose modification factors obtained are between 1.3 and 1.6. In order to ascertain if therapeutic gain could accrue from the use of pyrazinamide we have assessed its effect on the radiation response of mouse intestine using the crypt cell survival end point. The results shown in Figure 3 demonstrate that pyrazinamide does enhance radiation induced damage to crypt cells but to a lesser extent than seen with tumour. The radiation response curve is shifted down in a parallel fashion with a dose modification factor of between 1.0 and 1.2 over the radiation dose range studied.

To establish the mechanism responsible for the enhancement of tumour radiation response *in vivo* we have evaluated the radiosensitising and toxic effects of pyrazinamide on hypoxic cells *in vitro*. Figure 4 shows the radiation response of hypoxic CHO cells *in vitro* without drug or in the presence of either 10 mM misonidazole or 10 mM pyrazinamide. While misonidazole enhances radiation response of the cells (ER approx. 2.2), pyrazinamide has no observable effect. Figure 5 shows the hypoxic toxicity of either 10 mM misonidazole or 10 mM pyrazinamide over a 5 h incubation period. The results clearly show that over this time period misonidazole is cytotoxic to the hypoxic CHO cells while pyrazinamide has no effect. The absence of cytotoxicity towards hypoxic cells and lack of a marked effect of pyrazinamide on radiation response *in vitro* would suggest that the *in vivo* effect results either from a metabolite or from induced physiological changes in the animal. The results in Figure 6 show red blood cell (RBC) flow in SCCVII tumours as a function of time after pyrazinamide (0.5 mg $g^{-1}$ i.p.). A small increase in RBC flow occurs after drug administration. The maximum increase was 32% which was reached 40–45 min after drug administration. In addition to overall blood flow we have assessed the effect of pyrazinamide on the dynamic microregional heterogeneity of perfusion. Functional vasculature was demarcated at two instances in time separated by 20 min by injecting two fluorescent stains. For these experiments, pyrazinamide (or saline) was injected i.p. 45 min prior to the double staining procedure. Pretreatment with pyrazinamide reduces the amount of perfusion mismatch in tumour compared to saline treated controls (Figure 7). The percentage of mismatched vessels was scored in central and peripheral tumour regions as well as overall. The values obtained (± standard deviation) were (1) saline controls: central 14.5 (± 4.5), peripheral 7.8 (± 2.2), overall 10.3 (± 2.9); (2) pyrazinamide pretreatment: central 4.4 (± 2.8), peripheral 2.6 (± 0.9), overall 3.2 (± 1.5); simultaneous stain injection: central 1.6 (± 0.6), peripheral 1.1 (± 0.6), overall 1.3 (± 0.6).

**Figure 1** The effect of pyrazinamide (0.5 mg $g^{-1}$ i.p.) administered at various times before (−) or after (+) a fixed dose of X-rays. Cell survival was assessed 18 h after radiation. Shaded areas represent the effect of X-rays alone ± standard deviation. X-ray dose for EMT6 was 20 Gy, for LLC was 15 Gy.

**Figure 2** The effect of pyrazinamide (0.5 mg $g^{-1}$ i.p.) administered 45 min prior to radiation on the response of EMT6, LLC and SCCVII tumour models. Cell survival was assessed 18 h after radiation. Lines were fitted for doses greater than 10 Gy by linear regression analysis. (○) X-rays alone, (●) pyrazinamide 45 min prior to X-rays.

**Figure 3** Change in the response of mouse jejunum crypt cells to X-rays, by pyrazinamide. Saline or pyrazinamide (0.5 mg $g^{-1}$) was injected into non-tumour bearing mice 45 min prior to radiation. Crypt cell survival was measured 84 h later. (○) saline plus X-rays, (●) pyrazinamide plus X-rays. Mean ± 1 s.e. are shown.

**Figure 4** Radiation response of hypoxic CHO cells. (○) response to X-rays alone, (▲) X-rays plus 10 mM miso, (●) X-rays plus 10 mM pyrazinamide.

### Discussion

The results obtained show that pyrazinamide enhances the radiation response of three different murine tumour lines. Dose modification factors obtained for EMT6, LLC and SCCVII tumours are 1.6, 1.5 and 1.3 respectively. This enhancement of radiation effects seen in tumours is higher than the DMF of 1.0–1.2 obtained in mouse intestine, indicating the potential for therapeutic gain. Studies designed to indicate the mechanism of action of pyrazinamide suggest that changes in microregional blood flow may be involved.

**Figure 5** Cell survival as a function of time in hypoxia for CHO cells. (○) no treatment, (▲) in the presence of 10 mM misonidazole, (●) in the presence of 10 mM pyrazinamide.

**Figure 6** Relative RBC flow in SCCVII tumours as a function of time following administration of pyrazinamide (0.5 mg $g^{-1}$ i.p.). Data points are mean ± standard deviation.

**Figure 7** Effect of saline or pyrazinamide (0.5 mg $g^{-1}$ i.p.) on functional vasculature in 500–600 mg SCCVII tumours. Blood vessels outlined by either Hoechst 33342 or $DiOC_7(3)$ but not both are scored as mismatched. Both central and peripheral tumour regions were scored. Interval = O represents the background mismatch when both stains are injected simultaneously. Values represent mismatch scored in a minimum of 5 tumours for each treatment with 1,000 vessels being scored in each tumour, □ mismatch scored in peripheral regions, ▨ mismatch scored in central regions, ■ mismatch scored in tumour overall.

The radiation dose modification factors obtained for pyrazinamide in both tumours and intestine are comparable with these previously reported with nicotinamide (Horsman *et al.*, 1986, 1987). The *in vitro* data show that unlike the electron affinic radiosensitiser misonidazole, pyrazinamide displays no radiosensitising or toxic effects on hypoxic cells in culture. These comparisons were carried out at equimolar doses of both pyrazinamide and misonidazole. These findings suggest that pyrazinamide effects seen *in vivo* do not result from a direct radiosensitising action or from hypoxic cell cytotoxic effects of the parent molecule. This indicates that other *in vivo* processes are operating. Pyrazinamide may be metabolised *in vivo* to a product(s) which possesses direct radiosensitising and/or toxic properties towards hypoxic cells. Another possibility is that pyrazinamide (or a primary metabolite) could modify physiological parameters within the tumour resulting in a reduction in the radiobiologically hypoxic fraction. One key parameter in determining the number of hypoxic cells present in the tumour is oxygen delivery. Recent studies with nicotinamide have shown that a contributing factor to the radiosensitisation seen with this compound is microregional changes in tumour blood flow (Horsman *et al.*, 1989; Chaplin *et al.*, 1990); therefore, we have sought to establish if similar effects occur after pyrazinamide administration. Laser doppler flowmetry studies in the SCCVII tumour indicate that pyrazinamide produces a small (32%) but significant ($P < 0.05$) increase in blood flow in this tumour. However, this finding, although indicating that blood flow changes can occur after pyrazinamide, does not elucidate whether this reflects a small increase in flow in all tumour vessels, or a large increase in flow in a few vessels. This arises from the fact that although laser doppler flowmetry measures flow in a relative small volume (approx. 1 $mm^3$) compared to other techniques, such a volume still contains many blood vessels. The significance of this limitation would be particularly important if radiobiological hypoxia can occur from transient fluctuations in microregional blood flow and thus oxygen delivery. Such hypoxia is known to occur in experimental tumour systems (Chaplin *et al.*, 1986, 1987, 1990; Trotter *et al.*, 1989*b*; Minchinton *et al.*, 1990). Indeed, in the SCCVII tumour at a size of 500 mg, 8–10% of tumour vessels can open and close during a 20 min period (Trotter, 1989*b*). This latter study utilised a histological procedure which can provide a map of microregional blood flow (with resolution of single vessels) at two instances in time. The technique appears well suited to follow drug induced changes in functional vasculature (Trotter, 1989*c*; Chaplin, 1990; Zwi *et al.*, 1989). Using this technique with the SCCVII tumour, our present studies demonstrate that pyrazinamide has a marked effect on transient nonperfusion of vessels. Staining mismatch was reduced from 10.3% to 3.2% by pretreatment with pyrazinamide. This value is not significantly different from the background value obtained when the two fluorescent stains are injected simultaneously. These data provide evidence that at least part of the effect of pyrazinamide may be mediated by a reduction in 'acute' radiobiological hypoxia resulting from transient fluctuations in microregional perfusion. Similar results have been recently reported with nicotinamide (Chaplin *et al.*, 1990).

The modification of tumour hypoxia may not afford a complete explanation for the results obtained. This could be inferred from two factors in the survival curves shown in Figure 2. One is that survival levels at radiation doses of 15 Gy and above are higher than those expected from a totally aerobic population (Chaplin & Horsman, unpublished results). Secondly, the terminal slopes of the *in vivo* survival curves from pyrazinamide pretreated animals do not become parallel to the X-ray only curves, as would be expected if a residual hypoxic fraction remained. These findings could imply that some radiosensitising action (via a metabolite) is superimposed on the physiological actions of pyrazinamide. However, an alternative explanation which does not require any radiosensitising component to exist is that the remaining hypoxic cells after pyrazinamide pretreatment are diffusion limited 'chronically' hypoxic cells which possess a more sensitive radiation response than the acutely hypoxic cells (Franko & Sutherland, 1979).

Overall, the *in vivo* and *in vitro* results obtained with pyrazinamide bear a marked similarity to those previously reported for nicotinamide, thus suggesting common mechanisms of action. The fact that pyrazinamide is already clinically used in the treatment of tuberculosis (Weinstein, 1975) and the results presented here strongly suggest further studies with pyrazinamide and related analogues as modifiers of tumour response to ionising radiation.

This work was supported by a grant from the Medical Research Council of Canada. We would like to thank Ms Nancy Lepard, Ms Carrie Peters, Mr Hans Adomat and Mr Douglas Aoki for their excellent technical assistance.

### References

ADAMS, G.E., FOWLER, J.F., DISCHE, S. & THOMLINSON, R.H. (1976). Hypoxic cells sensitisers in radiotherapy. *Lancet*, **i**, 186.

ADAMS, G.E., CLARKE, E.D., FLOCKHART, I.R. & 8 others (1979). Structure-activity relationship in the development of hypoxic cell radiosensitisers I: Sensitising efficiency. *Int. J. Radiat. Biol.*, **35**, 133.

ADAMS, G.E. (1984). A conference preview. *Int. J. Radiat. Oncol. Biol. Phys.*, **10**, 1181.

BARENDSON, G.W., KOOT, C.J., VAN KERSON, G.R., BEWLEY, D.K., FIELD, S.B. & PAMELL, C.J. (1966). The effect of oxygen on the impairment of the proliferative capacity of human cells in culture by ionising radiation of different LET. *Int. J. Radiat. Biol.*, **10**, 317.

BROWN, J.M. & WORKMAN, P. (1980). Partition coefficient as a guide to the development of radiosensitisers which are less toxic than misonidazole. *Radiat. Res.*, **82**, 171.

BROWN, J.M. (1989). Hypoxic cell radiosensitisers: where next? *Int. J. Radiat. Oncol. Biol. Phys.*, **16**, 987.

CHAPLIN, D.J., SHELDON, P.W., STRATFORD, I.J., AHMED, I. & ADAMS, G.E. (1983). Radiosensitization in vivo: a study with an homologous series of 2-nitroimidazoles. *Int. J. Radiat. Biol.*, **44**, 387.

CHAPLIN, D.J., DURAND, R.E. & OLIVE, P.L. (1986). Acute hypoxia in tumors: implication for modifiers of radiation effects. *Int. J. Radiat. Oncol. Biol. Phys.*, **12**, 1279.

CHAPLIN, D.J., OLIVE, P.L. & DURAND, R.E. (1987). Intermittent blood flow in a murine tumor: radiobiological effects. *Cancer Res.*, **47**, 597.

CHAPLIN, D.J., HORSMAN, M.R. & TROTTER, M.J. (1990). The effect of nicotinamide on the microregional heterogeneity of oxygen delivery within a murine tumor. *J. Natl Cancer Inst.*, **82**, 672.

COLEMAN, C.N. (1988). Hypoxia in tumors: a paradigm for the approach to biochemical and physiological heterogeneity. *J. Natal Cancer Inst.*, **80**, 310.

COURTENAY, V.D. (1976). A soft agar colony assay for Lewis lung tumour and B16 melanoma taken directly from the mouse. *Br. J. Cancer*, **34**, 39.

DISCHE, S. (1989). Hypoxic cell sensitisers: clinical developments. *Int. J. Radiat. Oncol. Biol. Phys.*, **16**, 1057.

FRANKO, A. & SUTHERLAND, R.M. (1979). Radiation survival of cells from spheroids grown in different oxygen concentrations. *Radiat. Res.*, **79**, 454.

HENK, J.M., KINKLER, P.B. & SMITH, C.W. (1977). Radiotherapy and hyperbaric oxygen in head and neck cancer. *Lancet*, **ii**, 101.

HIRST, D.G. (1986). Oxygen delivery to tumors. *Int. J. Radiat. Oncol. Biol. Phys.*, **12**, 1271.

HIRST, D.G. & WOOD, P.J. (1989). Altered radiosensitivity in a mouse carcinoma after administration of clofibrate and bezafibrate. *Radiother. Oncol.*, **15**, 55.

HORSMAN, M.R., BROWN, D.M., LEMMON, M.J., BROWN, J.M. & LEE, W.W. (1986). Preferential tumor radiosensitization by analogs of nicotinamide and benzamide. *Int. J. Radiat. Oncol. Biol. Phys.*, **12**, 1307.

HORSMAN, M.R., CHAPLIN, D.J. & BROWN, J.M. (1987). Radiosensitization by nicotinamide in vivo: a greater enhancement of tumor damage compared to that of normal tissue. *Radiat. Res.*, **109**, 479.

HORSMAN, M.R., BROWN, J.M., HIRST, V.K. & 4 others (1988). Mechanisms of action of the selective tumor radiosensitizer nicotinamide. *Int. J. Radiat. Oncol. Biol. Phys.*, **15**, 685.

HORSMAN, M.R., CHAPLIN, D.J. & BROWN, J.M. (1989). Tumor radiosensitization by nicotinamide: a result of improved blood perfusion and oxygenation. *Radiat. Res.*, **118**, 139.

HORSMAN, M.R., EVANS, J.W. & BROWN, J.M. (1984). Enhancement of melphalan induced tumour cell killing by misonidazole: an interaction of competing mechanisms. *Br. J. Cancer*, **50**, 305.

JOHNSSON, G.G., KJELLER, E., PERO, R.W. & CAMERON, R. (1985). Radiosensitization effects of nicotinamide on malignant and normal mouse tissues. *Cancer Res.*, **45**, 3607.

MINCHINTON, A.I., DURAND, R.E. & CHAPLIN, D.J. (1990). Intermittent blood flow in the KHT sarcoma. Flow cytometric studies using Hoechst 33342. *Br. J. Cancer* (in the press).

MOORE, B.A., PALCIC, B. & SKARSGARD, L.D. (1976). Radiosensitizing and toxic effects of the 2-nitroimidazole Ro-07-0582 in hypoxic mammalian cells. *Radiat. Res.*, **67**, 459.

OLIVE, P.L., CHAPLIN, D.J. & DURAND, R.E. (1985). Pharmacokinetics, binding and distribution of Hoechst 33342 in spheroids and murine tumours. *Br. J. Cancer*, **52**, 739.

ROCKWELL, S.C., KALLMAN, R.F. & FAJARDO, L.F. (1972). Characteristics of a serially transplanted mouse mammary tumor and its tissue culture adapted derivatives. *J. Natl Cancer Inst.*, **49**, 735.

SHELDON, P.W. & HILL, S.A. (1977). Hypoxic cell radiosensitisers and local control by X-ray of a transplantable tumour in mice. *Br. J. Cancer*, **35**, 795.

SHEPHERD, A.P., RIEDEL, G.L., KIEL, J.W., HAUMSCHILD, D.J. & MAXWELL, L.C. (1987). Evaluation of an infrared laser-Doppler flowmeter. *Am. J. Physiol.*, **252**, G832.

TEICHER, B.A. & ROSE, C.M. (1984). Oxygen carrying perfluorochemical emulsion as an adjuvant to radiation therapy in mice. *Cancer Res.*, **44**, 428S.

THOMLINSON, R.H. & GRAY, L.H. (1955). The histological structure of some human lung cancers and the possible implications for radiotherapy. *Br. J. Cancer*, **9**, 539.

TROTTER, M.J., ACKER, B.D. & CHAPLIN, D.J. (1989*c*). Histological evidence for nonperfused vasculature in a murine tumor following hydralazine administration. *Int. J. Radiat. Oncol. Biol. Phys.*, **17**, 785.

TROTTER, M.J., CHAPLIN, D.J. & OLIVE, P.L. (1989*a*). Use of a carbocyanine dye as a marker of functional vasculature in murine tumours. *Br. J. Cancer*, **59**, 706.

TROTTER, M.J., CHAPLIN, D.J., DURAND, R.E. & OLIVE, P.L. (1989*b*). The use of fluorescent probes to identify regions of transient perfusion in murine tumors. *Int. J. Radiat. Oncol. Biol. Phys.*, **16**, 931.

WEINSTEIN, L. (1975). Drugs used in the chemotherapy of tuberculosis and leprosy. In *The Pharmacological Basis of Therapeutics*, 5th edition, Goodman, L.S. & Gilman, A. (eds) p. 1201. Macmillan: New York.

WITHERS, H.R. & ELKIND, M.M. (1970). Microcolony survival assay for cells of mouse intestinal mucosa exposed to radiation. *Int. J. Radiat. Biol.*, **17**, 261.

ZWI, L.J., BAGULEY, B., GAVIN, J.B. & WILSON, W.R. (1989). Blood flow as a major determinant in the antitumor action of flavone acetic acid. *J. Natl Cancer Inst.*, **81**, 1005.